# Scoping review and evidence mapping of interventions aimed at improving reproducible and replicable science: Protocol

**DOI:** 10.12688/openreseurope.16567.2

**Published:** 2024-07-10

**Authors:** Leonie A. Dudda, Magdalena Kozula, Tony Ross-Hellauer, Eva Kormann, René Spijker, Nicholas DeVito, Gowri Gopalakrishna, Veerle Van den Eynden, Patrick Onghena, Florian Naudet, Rita Banzi, Maddalena Fratelli, Monika Varga, Yuri Andrei Gelsleichter, Inge Stegeman, Mariska M. Leeflang

**Affiliations:** 1 Department of Otorhinolaryngology, Head and Neck Surgery, University Medical Center Utrecht, Utrecht, The Netherlands; 2 University Medical Center Utrecht Brain Center, University Medical Center, Utrecht, The Netherlands; 3 Faculty of Psychology and Educational Sciences, Methodology of Educational Sciences Research Group, KU Leuven, Leuven, Flanders, Belgium; 4 Institute of Interactive Systems and Data Science, Technische Universitat Graz, Graz, Styria, Austria; 5 Know-Center GmbH, Graz, Austria; 6 Cochrane Netherlands, Julius Center for Health Sciences and Primary Care, University Medical Center Utrecht, Utrecht, The Netherlands; 7 Amsterdam Public Health, Medical Library, Amsterdam UMC, Univ of Amsterdam, Amsterdam, The Netherlands; 8 Bennett Institute for Applied Data Science, Nuffield Department of Primary Care Health Sciences, University of Oxford, Oxford, England, UK; 9 Department of Epidemiology and Data Science, Amsterdam UMC, Amsterdam, The Netherlands; 10 Faculty of Health, Medicine & Life Sciences, Maastricht University, Maastricht, The Netherlands; 11 Research Coordination Office & RDM Competence Centre, KU Leuven, Leuven, Flanders, Belgium; 12 CHU Rennes, Inserm, Irset (Institut de recherche en santé, environnement et travail)-UMRS 1085, CIC 1414 [(Centre d'Investigation Clinique de Rennes)], Universite de Rennes, Rennes, Brittany, France; 13 Institut Universitaire de France, Paris, France; 14 Istituto di Ricerche Farmacologiche Mario Negri IRCCS, Milan, Italy; 15 Institute of Animal Sciences, Hungarian University of Agriculture and Life Sciences, Kaposvar, Hungary; 16 Department of Soil Science, Institute of Environmental Sciences, Hungarian University of Agriculture and Life Sciences, Gödöllő, Hungary

**Keywords:** Reproducibility, Replicability, Open Science, Transparency, Review

## Abstract

**Background:**

Many interventions, especially those linked to open science, have been proposed to improve reproducibility in science. To what extent these propositions are based on scientific evidence from empirical evaluations is not clear.

**Aims:**

The primary objective is to identify Open Science interventions that have been formally investigated regarding their influence on reproducibility and replicability. A secondary objective is to list any facilitators or barriers reported and to identify gaps in the evidence.

**Methods:**

We will search broadly by using electronic bibliographic databases, broad internet search, and contacting experts in the field of reproducibility, replicability, and open science. Any study investigating interventions for their influence on the reproducibility and replicability of research will be selected, including those studies additionally investigating drivers and barriers to the implementation and effectiveness of interventions. Studies will first be selected by title and abstract (if available) and then by reading the full text by at least two independent reviewers. We will analyze existing scientific evidence using scoping review and evidence gap mapping methodologies.

**Results:**

The results will be presented in interactive evidence maps, summarized in a narrative synthesis, and serve as input for subsequent research.

**Review registration:**

This protocol has been pre-registered on OSF under doi https://doi.org/10.17605/OSF.IO/D65YS

## Introduction

One of the scientific research objectives is to test theories, typically by accumulating knowledge derived from individual studies. This process is contingent on the reliability of previous findings that serve as the foundation for subsequent research. Essential prerequisites for trustworthy research findings are reproducibility — obtaining the same results when rerunning (parts of) a study using the same methodology, data and code — and replicability — obtaining the same outcome when repeating a study.

Unfortunately, research findings frequently cannot be reproduced due to, for example, inadequate information provided to rerun the experiment or analysis ( Alsheikh-Ali *et al*., 2011; Stodden *et al*., 2018). The EU report on the reproducibility of research results in EU framework programmes ( European Commission, Directorate-General for Research and Innovation, 2022) shows that many researchers rarely indicate to share their data, analysis code, or materials. This is problematic not only for the reproducibility of experiments but also for their ability to be replicated in other samples. Ensuring the reproducibility and replicability of scientific results is critical to the rigor and quality of research. When they are ignored, there is a risk that inaccurate, biased, or spurious results can gain undue attention in the literature leading to substantial research waste and flawed decision-making. Strategies to increase reproducibility and replicability have primarily focused on improving research transparency through various open science practices. These aim to ensure that the research process is documented and widely accessible so that it can be checked, critiqued, re-used, and built upon in future research. Ongoing initiatives, such as the ReproducibiliTEA network, community reporting guidelines and Open Science badges aim to facilitate the implementation of these practices in the research process. However, the extent to which these and other practices and interventions have been empirically investigated, and their actual impact on reproducibility and replicability, are unclear. Interventions to improve reproducibility/replicability outcomes have been conducted ( Ebersole *et al*., 2020; Hardwicke *et al*., 2021; Laurinavichyute *et al*., 2022; Obels *et al*., 2020) but have not been systematically assessed. We, therefore, aim to understand and provide an overview of the interventions that have been investigated for increasing the reproducibility and replicability of research and the documented barriers and facilitators in the process of creating more reproducible and replicable research using scoping review and evidence mapping methodology. For this review we use the definitions of replicability and reproducibility from Nosek *et al*. (2022) and European Commission's scoping and final report on reproducibility in research ( European Commission, Directorate-General for Research and Innovation, 2020 and European Commission, Directorate-General for Research and Innovation, 2022).

## Methods

This protocol was developed prior to the search and pre-registered on OSF under DOI 10.17605/OSF.IO/D65YS after including the final search strings. Title and abstract screening started in June 2023 based on a finished protocol. A well-constructed search strategy is the core of the systematic review. Due to the complexity of our search objectives, we revised the search string throughout the title and abstractscreening in order to improve its quality. The final search string will be published with this protocol. The final search string for the Medline database can be found in the section describing the search strategy. Potential changes to the protocol during the research process will be reported transparently. All additions to the protocol, such as data extraction sheets and data analysis plans, will be added to the OSF folder of this project under DOI 10.17605/OSF.IO/7EF5H whenever they are ready. The draft protocol has been reviewed by all authors. The protocol follows the Preferred Reporting Items for Systematic Reviews and Meta-Analyses Protocols (PRISMA-P) guidelines ( Moher *et al*., 2015).

### Design

Scoping review methodology is applied ( Arksey & O’Malley, 2005) since overviews regarding the evidence of the effect of interventions on reproducibility and replicability still need to be provided. We want to create an overview of the current evidence by systematically mapping available literature using the EPPI Reviewer software (EPPI Centre Software), thereby mapping inventions against reproducibility / replicability outcomes. By doing so, we aim to identify knowledge gaps and gain an overview of the characteristics of the current literature.

### Objectives

Our primary objective is to evaluate which interventions have been investigated for their effectiveness in improving reproducibility and replicability in science. These interventions can be applied on various levels (researchers, institutes, funders, publishers, editors, etc.). We specifically focus on Open Science practices defined broadly. The search strategy focuses on this objective.

The secondary objective is to investigate which drivers and barriers to the implementation and effectiveness of these interventions have been identified in these interventional studies.

**Figure 1.**
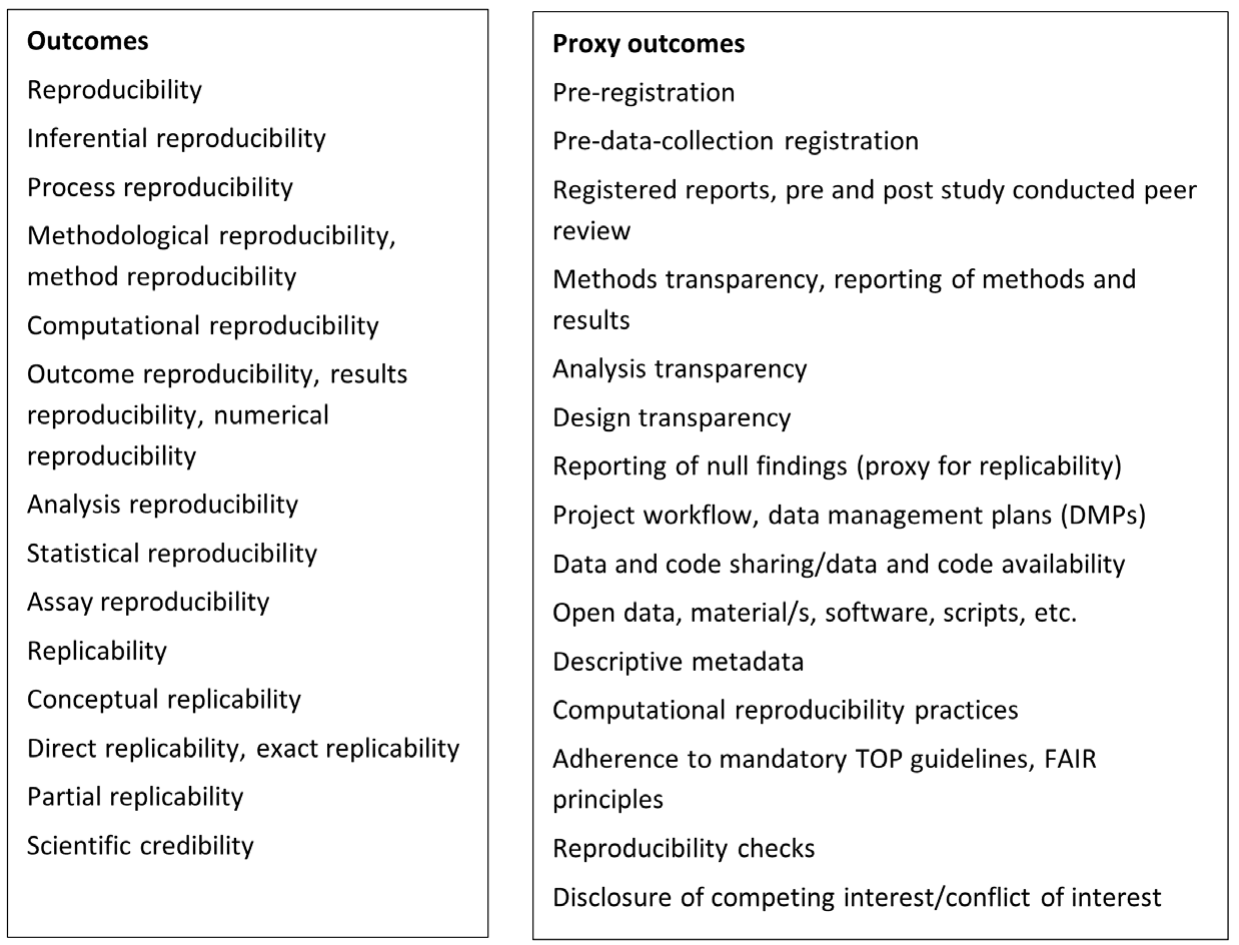
Reproducibility / replicability outcomes and proxy outcomes (Open Science practices believed to support reproducibility / replicability) to categorise data extraction; further explained in Appendix 1 (see data availability statement).

**Figure 2.**
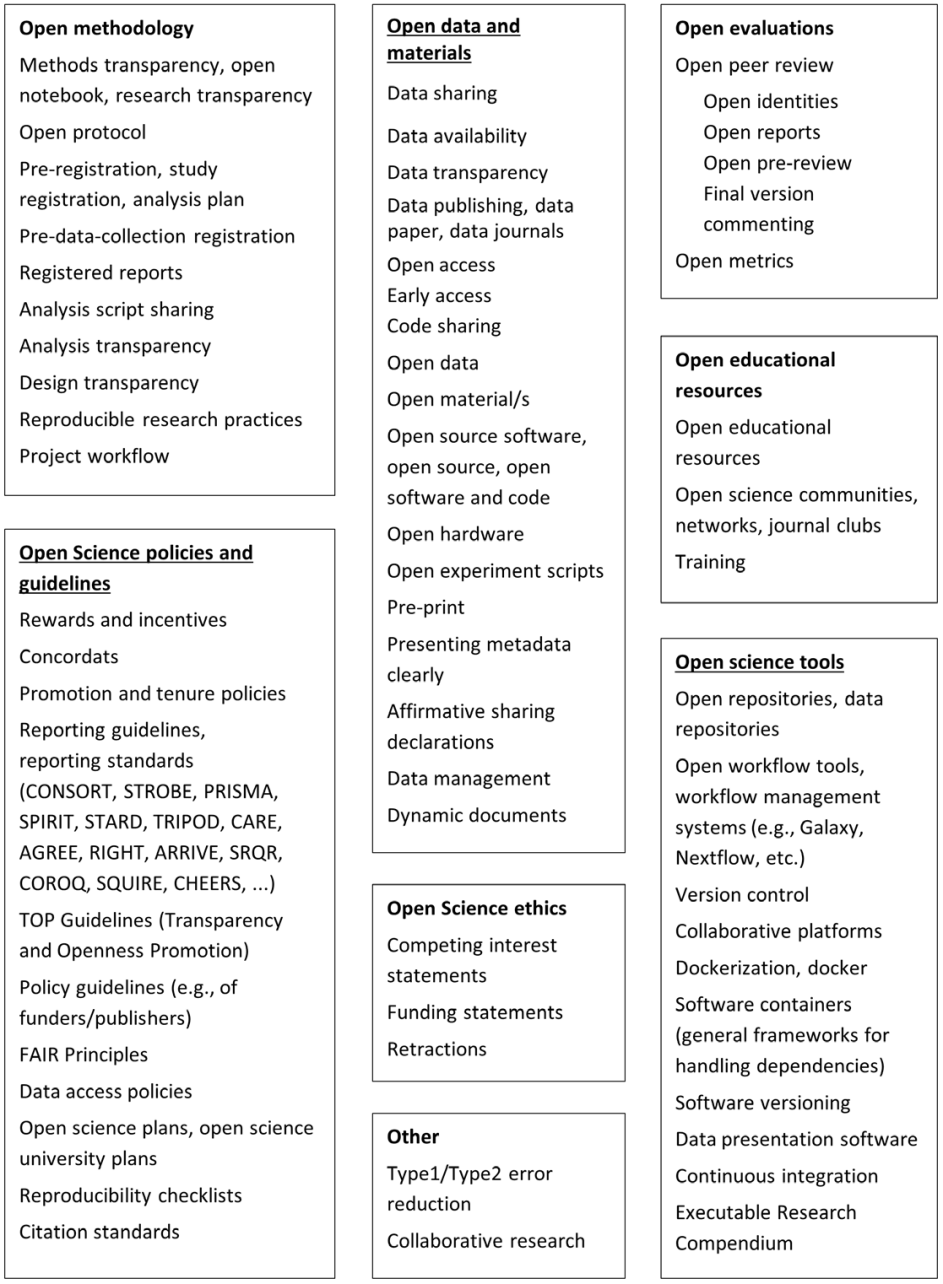
Categories of Open Science interventions used in the scoping review.

Based on the definitions we use for reproducibility / replicability, we created a list of Open Science interventions and reproducibility / replicability outcomes that will be investigated, which can be found in Figure 1 and Figure 2. These comprehensive lists which aim to capture all terminology for Open Science interventions and reproducibility / replicability outcomes used across disciplines, provide the framework for defining search terms and will form the basis of the categorisation of interventions and outcomes during data extraction. By adopting this list, we acknowledge that it might not be exhaustive as improving reproducibility in science is a continuous exercise, and there are many maturing ideas tackling the challenge ( Munafò *et al*., 2017).

### Disciplinarity

The notion of reproducibility and replicability varies across disciplines. Basing the categories of interventions and outcomes on broad definitions taken from multiple texts and searching ten databases to cover all disciplines, aims to cover that wide disciplinary variation. In addition, the protocol was developed by a multidisciplinary team and academic field is included as term for data extraction.

### Eligibility criteria

We will include studies evaluating the effectiveness of an intervention in promoting the reproducibility or replicability of scientific methods and findings and those additionally testing drivers and barriers of the process. Studies will be included independent of the specific approach applied to measure the outcome variable(s), such as using proxies or direct indicators of the outcomes.

Within the team we discussed how reproducibility may have been measured and came to the conclusion that some studies may have measured outcomes like preregistration, data sharing, code sharing, etc. Although data sharing and code sharing are conditional for reproducible research, simply looking at binary indications of whether data and code were shared is not necessarily a direct measure of reproducibility, as data and code can also be shared for other reasons. Therefore, we consider outcomes like rates of sharing data and code as proxies that could allow for reproducibility, rather than direct indications of reproducibility. So in addition to studies that explicitly evaluate interventions for reproducibility/replicability as their outcome, we will also consider studies that assess the effectiveness of interventions on proxy outcomes related to practices commonly claimed within the literature to support reproducibility/reproducibility to be in the scope of this review. The complete list of such proxies is included in Figure 1 and is based upon two criteria: the project team’s synthesis of key, widely cited prescriptive texts that provide a conceptual framework for the selection made ( Munafò *et al*., 2017; Stodden *et al*., 2016; European Commission, Directorate-General for Research and Innovation, 2020 and European Commission, Directorate-General for Research and Innovation, 2022) and the team’s evaluation which proxies would be measurable. Various Open Science practices included as interventions are also proxy outcomes for reproducibility / replicability if these references indicate them to likely enhance the opportunities for reproducibility / replicability.

Various study designs will be considered for inclusion in this research. To evaluate an intervention, comparisons must be made either between or within participants (see explanation below). Studies that do not have a comparator will be included only if the intervention is explicitly stated and the outcome variable measures reproducibility or replicability (for example, the prevalence of data sharing in journals with open science policies). These studies are included since they provide relevant information when compared with other studies (e.g., the prevalence of data sharing in journals without open science policy). Studies only investigating the prevalence of certain practices, such as data-sharing, are not included. Reviews will be included to apply the snowballing methodology to identify relevant literature, and since they might contain additional relevant information that is retrieved by the comparison and summary of primary studies. Reviews will be summarized separately to avoid duplication of primary studies in the evidence map.

Furthermore, we include studies that investigate facilitators and barriers in addition to evaluating the effectiveness of interventions on reproducibility and replicability (e.g., as moderator). When only drivers and barriers are assessed, for example, in the form of a survey or similar describing the opinions of multiple individuals without testing any intervention, these studies will be marked as such in the full-text screening phase. Our search was not specifically designed to find all barriers and facilitators though. Including these articles in the analysis is beyond this project's scope. Still, these papers are essential for understanding researchers' practices and decisions and compiling them might serve as a starting point for further projects. Article types such as position papers, study protocols, and other literature without primary data will not be considered. Additionally, we will search for and exclude retracted articles.

*Participants:* Researchers, institutes, funders, publishers, editors etc. in any field of research.

*Interventions:* Any intervention that aims to improve the reproducibility and replicability of science. These can be open science practices, such as the pre-registration of studies, open access publishing, data and code sharing or interventions to promote these open science practices, such as journal editorial or institutional policies, and implementation of guidelines or codes of conduct.

*Comparator:* Any comparator, including the absence of a comparator (e.g., pre-post comparison).

*Outcomes (qualitative and quantitative):* Reproducibility, replicability, and their proxies.

*Article types:* Articles, review articles and early access papers will be included.

*Study types:* Qualitative and quantitative interventional studies (e.g., pre-post or experimental designs).

### Search strategies

***Electronic searches***. At first, we identified key papers on the topic across various domains. This was be done by direct consultation with colleagues and reproducibility and open science experts within the OSIRIS consortium and its advisory board, for literature recommendations to fill any remaining gaps. The key papers were be expanded by citation coupling using Connected Papers. The team screened and discussed the new list, including an information specialist. The resulting set of documents both informed the building of a search query and validated the proposed search. The articles were added to a publicly available Zotero folder

Additionally, the team created a list of interventions and outcomes of interest (see Figure 1 and Figure 2). This list was informed by the team’s knowledge of the field, colleagues' consultation, and the key papers' screening. The search terms were defined based on the list and key papers. Before searching, the search strategy was checked by an external information specialist.

We will systematically search Medline, Embase, Web of Science, PsycINFO, Scopus, CAB Direct, Agris, PubAg, AGRICOLA and Eric. The proposed search will initially be developed for Medline and subsequently be translated to all databases. The search string for Medline can be found at the end of this section. No date or language restriction will be used. Grey literature will be searched separately by scanning websites of major policy actors (such as EC, Science Europe, EUA, NSF) for relevant interventional studies. After identifying relevant references, they will be used for reference and citation checking to determine potentially missed studies. The results from the searches will be collected in EndNote 20. Duplicate references will be removed with an in-built deduplication function of EndNote.

***Medline Search string***. (((data or code or workflow or practices or materials or notebook) adj2 (open or share or shared or sharing or preservation or stewardship)) or "open science" or ((computational or data or open or research or conclusion* or inferential or analytic or conceptual or direct or exact or statistical) adj3 (reproducib* or replicability or replicable)) or (research adj5 (transparen* or credib*))).ti,ab. or (reporting adj3 guideline*).ti.

(Clinical study/ or Case control study/ or Family study/ or Longitudinal study/ or Retrospective study/ or Prospective study/ or Cohort analysis/ or Comparative Study/ or (Cohort adj (study or studies)).mp. or (Case control adj (study or studies)).tw. or (follow up adj (study or studies)).tw. or (observational adj (study or studies)).tw. or (epidemiologic$ adj (study or studies)).tw. or (cross sectional adj (study or studies)).tw. or (comparative adj stud*).mp. or (("randomized controlled trial" or "controlled clinical trial" or "multicenter study" or "pragmatic clinical trial").pt. or non- randomized controlled trials as topic/ or interrupted time series analysis/ or controlled before-after studies/ or random*.ti,ab. or groups.ab. or (trial or multicenter or "multi center" or multicentre or "multi centre").ti. or (intervention? or effect? or impact? or controlled or control group? or (before adj12 after) or (pre adj5 post) or ((pretest or "pre test") and (posttest or "post test")) or quasiexperiment* or quasi experiment* or pseudo-experiment* or pseudoexperiment* or evaluat* or "time series" or time-point? or "repeated measur*" or ((experimental or empirical or qualitative) adj5 (study or studies))).ti,ab.)) not ((news or comment or editorial).pt. or comment on.cm.)

### Selection process

The title and abstract of all records identified through the respective search strategies will be screened for potential eligibility in duplicate independently. When there are disagreements, a third reviewer will be involved to resolve discrepancies in the selection of articles. The full-text manuscripts belonging to the titles and abstracts considered potentially eligible will then be read and selected for inclusion in duplicate independently. In this phase as well, disagreements will be solved by a third author.

### Data-extraction

The data extraction sheet, including extraction instructions and terminology definitions, will be developed in cooperation with the whole team. After extracting the first ten papers, the extraction sheet will be piloted and potentially adapted. Data extraction will be conducted in duplicate by two or more reviewers independently. Disagreements will be solved through discussion and additional consultation of a third reviewer. Data will be extracted from study documents and recorded in EPPI Reviewer across the items listed below. In case of insufficient data, we will attempt to contact the document’s authors for additional information. The following data will be extracted (if applicable):

a. Description of the publication
  - Title
  - Author(s)
  - Year of publication
  - Source (peer-reviewed, preprint, etc.)
  - Journal
  - Academic field
b. Type of publication
  - Systematic review, original research, research letter, etc.
c. Study design (in case the publication was a literature review, this item lists the study designs included in the review)
  - Research question
  - Comparative yes/no
  - Experimental yes/no
  - Qualitative design yes/no
  - Specific name of design
d. Description of the sample
  - Sample
  - Location/context
e. Interventions
  - Classification according to list of interventions in Figure 2
  - Stringency (mandatory, optional)
  - Implemented by
  - Target population
  - Stage of target process (before, during, after study conduct, after publication)
f. Outcome variables
  - Outcome variable (reproducibility, replicability, proxy)
  - Metric
  - Instrument
g. Drivers and barriers
  - E.g., time constraints that hinder the implementation of OS practices or programming knowledge reducing mistakes in shared code
h. Results
  - Answer to research question
  - Primary statistical results (effect size, p-value, CI, etc.)
  - Descriptives (means, percentages, etc.)
  - Meta-analytic results
  - Qualitative results
  - Additional results (e.g., drivers/barriers)

### Critical appraisal of individual sources of evidence

The aim of a scoping review is to identify and map the breadth of evidence available on a particular topic, field, concept or issue. Usually, coming to a formal statement about a certain effect or finding is not the aim, the strength or robustness of such a statement will also not be assessed. Furthermore, as a scoping review may cover multiple different study designs and article types, critical appraisal is not straightforward nor informative ( Munn *et al*., 2022; Peters *et al*., 2022). We will therefore not assess the risk of bias of each individual study included. We will, however, check for each article what the design was and whether a comparison was included (see data-extraction items).

### Data synthesis and analysis

All included studies will be summarized in a narrative synthesis. Quantitative analyses will be limited to listing numbers and/or proportions of studies assessing a certain intervention and outcome.

The evidence will be mapped according to intervention (rows) and reproducibility/replicability (columns) using the EPPI Reviewer software (EPPI Centre Software). Additionally, an adapted version of the categorization, as described by Davidson *et al*. (2022), will be applied to cluster different kinds of interventions.

The outcomes of reviews will not be included as a row in the evidence map but described separately. Evidence mapping will produce an interactive, searchable database of relevant studies, a list of knowledge gaps, and visualizations such as evidence atlases, heat maps, or descriptive plots. An interactive evidence map is a matrix of two variables, for example of interventions on the Y-axis and outcomes on the X-axis, providing a visual representation of the number of studies found on each combination of intervention and outcome. Since the intervention-outcome combinations for which no evidence exists leave a gap in the matrix, such a map is also called an evidence gap map. The map can be made interactive by enabling filtering or applying different sorting schemes. If possible, this will be done semi-automatically as well (using EPPI-reviewer, for example). These will aid in identifying gaps in the evidence and ways to address them. The exact layout of these figures will be determined after we have piloted the data extraction sheet.

### Study status

We are currently at the stage of title and abstract screening.

### Anticipated end of the study

The anticipated end date of the study is December 2023.

### Ethics and consent

Ethical approval and consent were not required.

## Data Availability

A list of definitions of terminology used in direct outcomes and proxy outcomes for the protocol has been published as supplementary materials in Open Science Framework: https://doi.org/10.17605/OSF.IO/BJR45. This material is available under the terms of the Creative Commons Zero “No rights reserved” data waiver (CC0 1.0 Public domain dedication).
