# Supplementary material for: Scoping review and evidence mapping of interventions aimed at improving reproducible and replicable science: Protocol
Source: Open Res Eur. 2024 Jul 10;3:179. Originally published 2023 Oct 18. [Version 2] doi: 10.12688/openreseurope.16567.2 (PMC11258544; doi:10.12688/openreseurope.16567.2)
Supplement: Peer review history

# Reviewer report: Reviewer response for version 2

**DOI:** 10.21956/openreseurope.19062.r42064

**Published:** 2024-07-22

**Authors:** Antica Culina

**Affiliations:** 1 Rudjer Boskovic Institute, Zagreb, Croatia

I approve this version of the article

Is the study design appropriate for the research question?

Yes

Are sufficient details of the methods provided to allow replication by others?

Yes

Is the rationale for, and objectives of, the study clearly described?

Yes

Are the datasets clearly presented in a useable and accessible format?

Yes

Reviewer Expertise:

Open science, open data, open codes, pre-registration, ecology, evolution

I confirm that I have read this submission and believe that I have an appropriate level of expertise to confirm that it is of an acceptable scientific standard.

# Reviewer report: Reviewer response for version 2

**DOI:** 10.21956/openreseurope.19062.r42066

**Published:** 2024-07-18

**Authors:** Rubén López-Nicolás

**Affiliations:** 1 Universidad de Murcia, Murcia, Region of Murcia, Spain

There are no new comments.

Is the study design appropriate for the research question?

Yes

Are sufficient details of the methods provided to allow replication by others?

Yes

Is the rationale for, and objectives of, the study clearly described?

Yes

Are the datasets clearly presented in a useable and accessible format?

Yes

Reviewer Expertise:

Research synthesis, reproducibility, meta-analysis

I confirm that I have read this submission and believe that I have an appropriate level of expertise to confirm that it is of an acceptable scientific standard.

# Reviewer report: Reviewer response for version 1

**DOI:** 10.21956/openreseurope.17882.r38293

**Published:** 2024-04-19

**Authors:** Lauren Supplee

**Affiliations:** 1 Independent Consultant, New York, NY, USA

Overall I think this is an interesting question and I look forward to hearing what they find. Similar to other interventions or policies, so many are theoretically selected but never empirically tested. It very well may be some of them have no effect at all and distract attention and resources from achieving the intended overall goal. My biggest concern is the very large bucket the authors are using to assess "impact". It appears they are intending to mix together qualitative and quantitative research, and research designs that do not measure impact. I see no concerns with including them in the search and description of the state of the field. I would recommend two actions. First, more clearly describe in the protocol which designs have strong internal validity to actually achieve their intended goal to assess the effectiveness of these interventions. Second, in the resulting findings then describe papers by whether they have the ability to isolate the effect of a specific intervention or core component of a policy, if they are suggestive of support for positive outcomes but need stronger designs, and complete gaps in the literature. That would be a much stronger contribution to the field, both for this paper and future work. 

 Regarding the dataset question, I couldn't tell from the information provided how the dataset will be shared for its own replication of this paper. The meta-data described seems appropriate but I couldn't tell where it will be housed and how it will support their own replication.

Is the study design appropriate for the research question?

Partly

Are sufficient details of the methods provided to allow replication by others?

Yes

Is the rationale for, and objectives of, the study clearly described?

Yes

Are the datasets clearly presented in a useable and accessible format?

Partly

Reviewer Expertise:

Evaluation of public policies and interventions and use of research in policymaking

I confirm that I have read this submission and believe that I have an appropriate level of expertise to confirm that it is of an acceptable scientific standard, however I have significant reservations, as outlined above.

# Reviewer report: Reviewer response for version 1

**DOI:** 10.21956/openreseurope.17882.r36821

**Published:** 2024-02-20

**Authors:** Olavo B. Amaral

**Affiliations:** 1 Federal University of Rio de Janeiro, Rio de Janeiro, Brazil

The paper describes the protocol of a scoping review of interventions to improve research reproducibility. While the goal of the review is extremely worthy, I found some of the details confusing (particularly with respect to the current state of the project and the use of the categories provided). I also believe the protocol would benefit from a clearer assessment of feasibility.

Major points:

 1. Perhaps the most obvious issue is that, from the paper, it is not really clear at what point the project is. On the contrary, contradictory verb tenses across the manuscript make this quite confusing.

 The Methods section starts out with “this protocol will be pre-registered on OSF after including the final search strings”, and then immediately gives a DOI for the preregistration. It then proceeds with “title and abstract screening started in June 2023”, even though (a) the section describing screening in the Methods is written in the future tense and (b) the text has just stated that the final search strings were yet to be developed.

 These contradictions come back in later section, where the Methods state that “the team will create a list of interventions and outcome of interest” and then cites Figure 1 and Figure 2 (in which this list seems to already have been developed). And then again when they state that “the search terms will be defined based on the list and key papers” and that “the proposed search will initially be developed for Medline” and then follows up with the complete MedLine string.

 I have no problem with part of the work having been performed, or with preliminary searches being carried out – such pilots tend to be useful for refining the protocol. But the authors have to be clear about what has already been performed and what is yet to be done. This is not the case in the current state of the manuscript and should be thoroughly revised.

 2. Another confusing issue is the meaning of the lists in Figure 1 and Figure 2. Are the concepts listed meant to be used as inclusion criteria in the systematic review (e.g. are these the outcomes and types of interventions that qualify for inclusion)? As concepts reflected in the search strings (although this does not seem to be the case in the provided string)? As categories for grouping interventions and outcomes in the review? I really could not understand, and the lack of legends in the figures (as well as their disconnect from the text that follows their appearance) doesn’t help.

 My further comments on the lists are naturally limited by not understanding what the terms are intended to be used for. That said, I would note that they are quite dense in technical and/or vague terms that are not precisely defined. Some examples:

 - What is meant by “process reproducibility”, and how does it differ from “method reproducibility”?

 - “Descriptive metadata” refers to that? Studies? Datasets?

 - What fits under very vague terms such as “methods transparency”, “reproducible research practices” or “training”?

 If these lists are indeed important for the review process, these and other terms would benefit from a more precise definition and/or references (even if this requires an appendix or supplementary material). In fact, specific guidelines cited (e.g. TOP, FAIR principles) likely require references that have not been included anyway.

 Also, if the categories are meant to be used as inclusion criteria (once again, I don’t understand if this is the case), I’d question some of the items in the list. “Scientific credibility” as an outcome (unless it is defined in a very specific way) would lead to the inclusion of lots of studies on public perception of science that are not about reproducibility *per se*. And although disclosure of competing interests is important, I’m not sure I’d consider it as a proxy outcome for reproducibility – this has more to do with general research integrity and will probably lead to the inclusion of a literature that does not deal directly with reproducibility.

 Finally, I note that some concepts are included both as proxy outcomes and interventions (e.g. “Methods transparency”). Although I can see why this could be the case (e.g. one could test a specific intervention to see whether it improves methods transparency, but also test whether something that clearly improved methods transparency improves reproducibility), it leads to a strange impression of circularity. It’s worth thinking whether there might be a better taxonomy to use in order to avoid this. If there isn’t, the authors should at least note this apparent contradiction explicitly and explain why some things can be both interventions and outcomes.

 3. I’m a bit concerned with the feasibility of the review. With the snowballing approach, the very general definitions of interventions and outcomes, the large number of databases and the inclusion of grey literature, this could turn out to be a lot of work. Do the authors have any estimate of this workload, as well as the expected time to perform it, given the size of the team?

 In order to evaluate the protocol’s feasibility, it would be useful if the authors could provide an estimate of the number of articles retrieved by the search (perhaps using their included search string), of the number of included articles (perhaps based on the inclusion rate of a pilot set of article), the number of people involved in each step and their expected deadline for concluding the review. As a reviewer, I would feel much more comfortable accepting the protocol for publication after looking at this data, in order to check whether it seems feasible and likely to be followed and become a systematic review in the future.

 4. The interventions to improve reproducibility, outcomes to measure it, and in fact the very notion of “reproducibility” and its relative importance are likely to vary a lot from one scientific field to another (see Leonelli, S. (2018)[Ref-1] and others). This discussion, however, is almostly completely lacking in the protocol. The review seems set to include interventions in any field of research, but will interventions really be commensurable between them? Will any attempt be made to summarize interventions in a discipline-specific manner? This topic likely deserves some attention in the introduction and in the description of the analysis.

Minor points:

 Abstract:

 - Instead of “combat the reproducibility crisis”, perhaps the authors should use a less charged term such as “improve reproducibility”. There is discussion of whether the “crisis” narrative is appropriate, and it probably does not apply equally to all scientific fields. As it seems unnecessary that there is a crisis for interventions to work, it might be worth removing the term here.

 - It is unclear what the term “semi-automated” refers to. Does it refer to the title and abstract screening? If so, perhaps it should be “semi-automatedly”? I also note that there is no mention of automating screening in the methods, so this should be checked.

 - Can the authors be more specific on what constitutes an “interactive evidence map”.

 Introduction:

 - There seems to be a contradiction in the initial definitions of reproducibility. The first definition given for reproducibility is in line with the one by the US National Academy of Sciences, (e.g. obtaining the same results with the same data). The next sentence, however, mentions “rerunning experiments”, which seems to refer to what would be called replicability in this framework – unless it is talking about computational experiments, which would not be intuitive for most readers. The authors then start using “replicability” as well, but don’t define the concept. They later mention using the definition by Nosek et al. (2022) ^2^ and the European Commission, but only in the Methods section - if that is the case, shouldn’t these references be cited upfront in the introduction?

 - Can the authors provide more examples of initiatives to integrate open practices in the research process besides ReproducibiliTea (which I would argue is only very indirectly linked to this purpose and would not be the first example that comes to my mind)?

 Methods:

 - Please define the abbreviation in “T&A screening” (it probably means title and abstract, but this is not specified”.

 - PRISMA-ScR is a reporting guideline for scoping reviews, so I’m not sure it applies to protocols. There is a PRISMA extension for protocols Moher et al.,(2015) [Ref-3]. Shouldn’t this be used instead?

 - The “design” section says little about the design. Once again, what do the authors mean by “systematically map available evidence”. It would be useful if they at least mention in which dimensions they plan to be mapping it.

 - *“In addition to studies that explicitly evaluate interventions for reproducibility/replicability, we will also consider studies that assess the effectiveness of interventions related to practices commonly claimed within the literature to support reproducibility/reproducibility to be in the scope of this review”*

 The distinction is not clear in this sentence: in the way this is written, isn’t the second category contained in the first? The rest of the paragraph seems to imply that the distinction has to do with using actual reproducibility/replicability vs. indirect proxies (e.g. transparency) as the outcome. However, this is not what’s written in this sentence, which by itself does not make sense to me.

 - “ *Comparisons must be made either between or within subjects*”. What counts as a “subject” here? Is it a researcher? Could it be an article, an institution or a journal? Although this information seems to be included in “participants” later, what is meant by “subject” should be stated before this sentence for it to make sense.

 - “ *This will be done by direct consultation with colleagues and reproducibility and open science experts for literature recommendations to fill any remaining gaps.” *How will these experts be defined and how many of them are expected to be consulted?

 - Ten papers sound like a very small sample to develop the extraction sheet. I’d assume that the types of studies may vary a lot in this review; thus, I’d recommend that a larger pilot sample is used in order to have a better idea of what can come up and guide the extraction process.

 - “ *Classification according to list of interventions in appendix A”.*

 What is Appendix A? This seems to refer to the figures (which were an Appendix in the OSF protocol), but the paper itself mentions no appendices.

 -  The “results” field in the data extraction section seem to go beyond what would be analyzed in a scoping review, mentioning extraction of primary statistical results, meta-analytic results, etc. Will this be analyzed in the current review? Will it be used in later steps? Please clarify.

Is the study design appropriate for the research question?

Yes

Are sufficient details of the methods provided to allow replication by others?

Partly

Is the rationale for, and objectives of, the study clearly described?

Yes

Are the datasets clearly presented in a useable and accessible format?

No

Reviewer Expertise:

Metaresearch

I confirm that I have read this submission and believe that I have an appropriate level of expertise to confirm that it is of an acceptable scientific standard, however I have significant reservations, as outlined above.

## References

[1] : Rethinking Reproducibility as a Criterion for Research Quality.36: 10.1108/S0743-41542018000036B009 129-146 10.1108/S0743-41542018000036B009

[2] : Replicability, Robustness, and Reproducibility in Psychological Science. *Annu Rev Psychol* .2022;73: 10.1146/annurev-psych-020821-114157 719-748 10.1146/annurev-psych-020821-114157 34665669

[3] : Preferred reporting items for systematic review and meta-analysis protocols (PRISMA-P) 2015 statement. *Syst Rev* .2015;4(1) : 10.1186/2046-4053-4-1 1 10.1186/2046-4053-4-1 25554246 PMC4320440

# Response

**Published:** 2024-04-04

**Authors:** Veerle Van den Eynden

Thank you very much for the review of our manuscript and for the very helpful comments you have made. We have addressed them all in the revised version of the manuscript that we are submitting now, and explain here the changes made. **Major comments:** 1. We have revised and adjusted the verb tenses throughout the manuscript to make clear that at the time of submitting the protocol the OSF registration had been done, the interventions/outcome lists had been finalised and published and the search string had been finalised. 2. We have explained better what the lists in Figure 1 & 2 mean, how they were developed and which purpose they serve; have expanded the captions for the figures and included a list of definitions / explanations with references for the outcomes, as Appendix to the manuscript.  We agree with you that terms such as "scientific credibility" and "disclosure of competing interests" are too broad as terms to be useful proxy outcomes. Whilst they were used in the search string, during screening of resulting studies we did not include all scientific credibility studies and also removed competing interests studies. This will be explained in the paper describing the results of the scoping review. We have explained in Eligibility criteria why it is that some concepts can be both proxy outxomes and interventions. 3. By now the scoping review has been finalised and we will soon publish the results which will indicate the number of articiles retrieved, screened, etc. Whilst  due to the broad scope of our approach, it was indeed a large undertaking, that does not mean the study was not feasible. We defined the protocol that we deemed was needed to assess the evidence & gaps in interventions tested that we need for our project. The results paper will describe the complexity / feasibility of the protocol and how we achieved the results. 4. We have included a section on Disciplinarity in the manuscript. **Minor points:** Abstract: We have made the siggested changes and explained what interative evidence maps are in the Data synthesis & analysis section. Introduction: We have brought the Definitions used into the Introduction and made sure they align. We have added more examples of initiatives.  Methods: Corrections made, and the reporting guideline indeed changed to PRISMA-P. Design is explained better and proxy outcomes are explained. Subjects has been changed to Participants. We explained who the experts are.  Extraction sheet: We had already done the testing of the extraction sheet by the time this review was done. Thank you for the suggestion though. Regarding the results fileds in the data extraction sheet, we extracted key design characteristics for descriptive analysis but not for meta-analysis for the scoping review. This information can, however, be potentially explored in further project activities.      

# Reviewer report: Reviewer response for version 1

**DOI:** 10.21956/openreseurope.17882.r36826

**Published:** 2024-02-20

**Authors:** Rubén López-Nicolás

**Affiliations:** 1 Universidad de Murcia, Murcia, Region of Murcia, Spain

In this manuscript, the authors present a protocol for a scoping review of the existing evidence regarding interventions aimed at enhancing reproducibility and replicability. I find the topic highly compelling and pertinent; shedding light on this issue is undoubtedly necessary.

 However, I have a few comments, mainly concerning the clarity and presentation of the issue at hand:

 While the study focuses on reproducibility and replicability, exploring various aspects of both (as indicated in Fig. 1, Outcomes), the introduction explicitly defines only reproducibility. Additionally, although both reproducibility and replicability are later referenced in the introduction, a distinction between the two is absent in this section. Improving the introduction by providing clearer definitions for the various concepts that will be central to this work would enhance its overall clarity and understanding. While two references are indeed mentioned later in the 'Definitions' section, an earlier clarification would be very helpful.

 On the other hand, I also noticed a lack of a brief discussion of the state of the art of the topic at hand in the introduction. The studies of interest seem to be papers evaluating the effectiveness of interventions to improve reproducibility and replicability. It might be useful to mention papers in this regard in the introduction, commenting on what their approach was, what intervention they evaluated, what they found, etc. It would help to put the focus on the specific topic.

 The methodology, arguably the most relevant section of the protocol, is adequately described in my opinion. The iterative approach to the electronic search and the design of the data extraction sheet also seems to me to be adequate. It might be relevant to mention whether any kind of quantitative synthesis is planned if the conditions become suitable and what those conditions would be. But there may not be any intention of this being a scoping review.

 Best of luck in the progress of this interesting work.

Is the study design appropriate for the research question?

Yes

Are sufficient details of the methods provided to allow replication by others?

Yes

Is the rationale for, and objectives of, the study clearly described?

Yes

Are the datasets clearly presented in a useable and accessible format?

Yes

Reviewer Expertise:

Research synthesis, reproducibility, meta-analysis

I confirm that I have read this submission and believe that I have an appropriate level of expertise to confirm that it is of an acceptable scientific standard, however I have significant reservations, as outlined above.

# Response

**Published:** 2024-04-04

**Authors:** Veerle Van den Eynden

Thank you very much for your review of our mansucript and for the constructive comments / suggestions made. We are explaining here how we have addressed those in the revised manuscript that we are submitting now. *While the study focuses on reproducibility and replicability, exploring various aspects of both (as indicated in Fig. 1, Outcomes), the introduction explicitly defines only reproducibility. Additionally, although both reproducibility and replicability are later referenced in the introduction, a distinction between the two is absent in this section. Improving the introduction by providing clearer definitions for the various concepts that will be central to this work would enhance its overall clarity and understanding. While two references are indeed mentioned later in the 'Definitions' section, an earlier clarification would be very helpful.* We have made the definitions of reproducibility & replicability clearer in the introduction, and moved the Definitions that we use also into the Introduction. 

*On the other hand, I also noticed a lack of a brief discussion of the state of the art of the topic at hand in the introduction. The studies of interest seem to be papers evaluating the effectiveness of interventions to improve reproducibility and replicability. It might be useful to mention papers in this regard in the introduction, commenting on what their approach was, what intervention they evaluated, what they found, etc. It would help to put the focus on the specific topic.* We have expanded the state of the art in the Introduction and included literature references. *The methodology, arguably the most relevant section of the protocol, is adequately described in my opinion. The iterative approach to the electronic search and the design of the data extraction sheet also seems to me to be adequate. It might be relevant to mention whether any kind of quantitative synthesis is planned if the conditions become suitable and what those conditions would be. But there may not be any intention of this being a scoping review.* We have explained which quantitative analysis is planned and expanded the Data synthesis and analysis section.

# Reviewer report: Reviewer response for version 1

**DOI:** 10.21956/openreseurope.17882.r35721

**Published:** 2023-11-13

**Authors:** Antica Culina

**Affiliations:** 1 Rudjer Boskovic Institute, Zagreb, Croatia

The manuscript is a study protocol identifying the rationale and methodology for conducting evidence mapping of interventions for increasing reproducibility and replicability. I find the study very interesting and relevant given the current need to identify whether and how open science improves credibility and impact of research. Overall, methodology is well described and developed for this stage of the research. I only have several relatively minor comments/suggestions for the MS.

 My main comment is about the scope of the mapping which is somewhat not consistently described through the MS. This is relevant for two aspects:

1. Reading the Background, I’ve got the impression this work will look into the strength of the evidence (the last sentence of the Background). However, it is mapping the literature that contains the evidence, rather than calculating some effect sizes from the evidence. I think it would be useful that the text reflects this. E.g. last sentence of the Background could be something like ‘ Much of the literature has looked at the evidence that xxx but this literature has not been systematically mapped.’ Similar, the sentence of the Introduction: ‘ However, the extent to which these and other practices …. Etc’ gives the impression that some effect sizes will be calculated from the obtained evidence.
2. The title and the aims are about any practices that can improve reproducibility and replicability. However, the Interventions that are listed in the Fig 2 are only about Open practices.

Some minor comments:

1. In the Background section of the abstract, please consider adding ‘/replicability’ to read ‘.. combat the reproducibility/replicability crisis.’ While I understand that reproducibility crisis is the original term that was used to describe non-replication of study results, maybe it would be easier for the reader to have both terms mentioned in the Background section.
2. I do not understand why are the listed proxy outcomes (Fig 1) in fact proxies for the main outcomes. Could you explain the reasoning behind these?
3. Fig 2: Under ‘ Other’  it is unclear why these exact terms were chosen? Why not p-hacking reduction, or HARKING reduction?
4. The MS could better highlight that mapping the barriers and facilitators is not fully systematic, as it will be done based on the literature obtained via the main search (which is to identify practices and their potential impacts), and not by an interdependent search of the literature on facilitators/barriers.
5. Could you please provide a very brief explanation on why Risk of Bias will not be assessed?
6. Maybe change the date for the ‘Anticipated end of the study’?

Is the study design appropriate for the research question?

Yes

Are sufficient details of the methods provided to allow replication by others?

Yes

Is the rationale for, and objectives of, the study clearly described?

Yes

Are the datasets clearly presented in a useable and accessible format?

Yes

Reviewer Expertise:

Open science, open data, open codes, pre-registration, ecology, evolution

I confirm that I have read this submission and believe that I have an appropriate level of expertise to confirm that it is of an acceptable scientific standard, however I have significant reservations, as outlined above.

# Response

**Published:** 2024-04-04

**Authors:** Veerle Van den Eynden

Thank you very much for your review of our protocol and for the constructive comments. We have made the necessary changes in the manuscript that we are resubmitting now and explain here how we addressed your comments. **Major comments:** 1. We have explained this better in the Introduction 2. We have chenged the Title, Aim & Objectives to indicate the focus on Open Science practices. **Minor comments:** 1. We have harmonised the focus on reproducibility/replicability throughout the text.  2. We have explained the proxy outcomes better in the text on Eligibility 3. P-hacking reduction is the same as type2 error, so is included in the category "Type1/Type2 error reduction". Harking reduction is addressed in "Preregistration / analysis plan". 4. We have indicated in Eligibility criteria that the search was not specifically designed to find all barriers & facilitators. 5.  We explained why risk of bias is not assessed in the Critical appraisal section. 6. We have updated the date.
